# Complete circular genome sequences representing prevalent sub-lineages of clinical *Listeria monocytogenes* isolates from Germany

**DOI:** 10.1128/mra.00145-26

**Published:** 2026-04-23

**Authors:** Sabrina Wamp, Antje Flieger, Sven Halbedel

**Affiliations:** 1 FG11 Division of Enteropathogenic Bacteria and Legionella, German Consultant Laboratory for Listeria, Robert Koch Institute 9222, Wernigerode, Germany; 2 Institute for Medical Microbiology and Hospital Hygiene, Otto von Guericke University Magdeburg 9376, Magdeburg, Germany; Fluxus Inc., Sunnyvale, California, USA

**Keywords:** invasive listeriosis, molecular surveillance, whole genome sequencing, reference genome

## Abstract

We here present 27 closed genome sequences of clinical *Listeria monocytogenes* isolates representing 26 prevalent phylogenetic sub-lineages detected in Germany within recent years. This information may facilitate analysis of ongoing and future outbreaks and provides the basis for in-depth studies of outbreak strains and their genetic characteristics.

## ANNOUNCEMENT

*Listeria monocytogenes* is the causative agent of listeriosis, a foodborne disease that can lead to severe invasive infections (1). Here, we provide 27 closed genomes of clinical *L. monocytogenes* isolates, which were collected from German listeriosis patients between 2016 and 2023 and sent to the German Consultant Laboratory for *Listeria* for subtyping. These genomes represent a selection of the most prevalent *L. monocytogenes* sub-lineages causing listeriosis in Germany.

Selected isolates were thawed from glycerol stocks of the strain collection of the Consultant Laboratory and cultivated in 3 mL brain heart infusion broth with shaking (250 rpm) overnight at 37°C. Genomic DNA was extracted with the GenElute Bacterial Genomic DNA Kit (Sigma-Aldrich), and the quality and concentration of the DNA were determined with a NanoDrop spectrophotometer (Thermo Fisher Scientific) and the Qubit fluorometer (Thermo Fisher Scientific). Short-read sequencing data were taken from subcultures described in previous work (2) or generated by library construction using the Nextera XT DNA Library Prep Kit (Illumina) and subsequent sequencing on a NextSeq sequencer (Illumina) in paired-end sequencing mode (2 × 150 bp). The read files were analyzed using an in-house quality control pipeline that includes, but is not limited to, QCumber-2 v2.3.0 (3), FastQC v0.11.5 (4), and Kraken v0.10.6 (5). Libraries for long read sequencing were constructed with the Rapid barcoding kit (SQK-RBK004 / SQK-NBD114.24, Oxford Nanopore Technologies) and subsequently sequenced on a FLOMIN106D or FLOMIN114 flow cell on a GridION device (Oxford Nanopore Technologies) with ont-guppy-for-gridion base-calling models in the super-accurate mode, a Q-score threshold of 10, and adapter trimming. Filtlong v0.2.1 (6) was used to refine the obtained long reads based on the corresponding Illumina reads as an external reference, while also keeping only reads with a minimal length of 3,000 bp. For filtering, the number of target bases was set to 290,000,000 bp, resulting in a ~100- fold coverage. The filtered long reads and the Illumina short reads were used in a hybrid assembly with Unicycler v0.4.8 (7) to obtain a closed genome sequence. Ring closure was verified in Geneious Prime 2025.0.2 (Dotmatics), which was also used to rotate the genome sequences to the first nucleotide of the characteristic *L. monocytogenes* origin of replication (8). The genomes were then annotated with the Prokaryotic Genome Annotation Pipeline (PGAP) v6.8 from the National Center for Biotechnology Information (NCBI) (9).

The 27 closed *L. monocytogenes* genomes represent 26 different multilocus sequence typing (MLST) sequence types (STs), reflecting different phylogenetic sub-lineages. All strains carry the *Listeria* pathogenicity island 1 (LIPI-1), and selected isolates additionally possess LIPI-3 and/or LIPI-4. Twenty-three of the clinical isolates were assigned to outbreak clusters, while four strains represented sporadic cases. Genome sizes vary depending on the number of inserted prophages (according to PHASTER [10]). Several isolates contain previously described plasmids carrying genes involved in heavy metal transport (11, 12) (Table 1).

**TABLE 1:** Key characteristics of the genomes sequenced in this study

Isolate ID	18-00200	18-01610	18-02301	18-03000	18-03367	18-04006	19-05461	19-05633	19-07356	19-07396	19-07613	20-01331	20-03334	­
Outbreak	Beta1	Phi4	Alpha5	Gamma6a	Ny5a	Gamma5a	Lambda5a	Tau7	Ypsilon1a	Eta4	Gamma7	Tau8	Psi1	
Source	clinical isolate	clinical isolate	clinical isolate	clinical isolate	clinical isolate	clinical isolate	clinical isolate	clinical isolate	clinical isolate	clinical isolate	clinical isolate	clinical isolate	clinical isolate	
Year of isolation	2018	2018	2018	2018	2018	2018	2019	2019	2019	2019	2019	2020	2020	
GenBank accession no.	CP169662	CP169664	CP183327	CP169693	CP169665	CP169666	CP169671	CP169672	CP169695	CP169674	CP169675	CP169676	CP169699	
ENA accession no.	SAMEA114338268	SAMEA114338283	SAMEA114332999	SAMEA114338287	SAMEA6800951	SAMEA114333032	SAMEA114339229	SAMEA114338331	SAMEA114338343	SAMEA114338345	SAMEA114338350	SAMEA114338358	SAMEA114338389	
Sequencing method	Oxford Nanopore MinION, Illumina MiSeq	Oxford Nanopore MinION, Illumina MiSeq	Oxford Nanopore MinION, Illumina MiSeq	Oxford Nanopore MinION, Illumina MiSeq	Oxford Nanopore MinION, Illumina MiSeq	Oxford Nanopore MinION, Illumina MiSeq	Oxford Nanopore MinION, Illumina NextSeq	Oxford Nanopore MinION, Illumina NextSeq	Oxford Nanopore MinION, Illumina MiSeq	Oxford Nanopore MinION, Illumina MiSeq	Oxford Nanopore MinION, Illumina MiSeq	Oxford Nanopore MinION, Illumina MiSeq	Oxford Nanopore MinION, Illumina NextSeq	
Accession no. for long-read raw data	ERX11576225	ERX11576227	ERX11576228	ERX11576230	ERX12726491	ERX11576231	ERX11576236	ERX11576237	ERX11576239	ERX11576240	ERX11576241	ERX11576242	ERX11576245	
Number of long reads	76,666	93,901	98,405	214,503	48,318	264,541	73,775	79,197	563,061	96,212	68,341	268,750	523,028	
N50 long reads	11,610	9,654	10,675	17,937	15,133	14,903	11,869	10,832	14,758	10,982	10,585	13,411	12,903	
Accession no. for short-read raw data	ERX11412217	ERX11412232	ERX11405822	ERX11412236	ERX4076975	ERX11405855	ERX11414494	ERX11412280	ERX11412292	ERX11412294	ERX11412299	ERX11412307	ERX11412338	
Number of short reads	1,732,788	3,051,586	2,090,022	1,806,692	1,916,996	1,677,342	4,917,118	4,628,822	1,987,750	2,189,770	1,622,482	1,792,946	3,233,208	
Genome size (bp)	3,033,395	2,917,851	3,007,044	2,955,798	3,034,335	2,934,767	2,972,356	2,940,533	2,941,803	2,968,280	3,011,468	2,908,639	2,982,651	
GC content (%)	38	38	38.1	37.9	37.9	38	38	38	38	38	37.9	38	38	
No. of protein-coding genes	2,962	2,788	2,926	2,960	2,939	2,804	2,925	2,842	2,912	2,861	2,927	2,823	2,948	
No. of rRNA operons	6	6	6	6	6	6	6	6	6	6	6	6	6	
No. of tRNA genes	67	67	67	67	67	67	67	67	67	67	67	67	67	
Pathogenicity island(s)	LIPI-1	LIPI-1, LIPI-4	LIPI-1, LIPI-3	LIPI-1	LIPI-1	LIPI-1, LIPI-4	LIPI-1	LIPI-1	LIPI-1, LIPI-3	LIPI-1, LIPI-3	LIPI-1	LIPI-1	LIPI-1, LIPI-3	
Plasmid (GenBank accession no.)			CP183328 (32,051 bp)	CP169694 (86,632 bp)					CP169696 (58,527 bp)				CP169700 (69,602 bp)	
PCR serogroup	IIb	IVb	IIb	IIa	IVb	IIb	IIa	IIa	IVb	IVb	IIa	IIa	IVb	
Intact prophages (10)	3	0	2	2	2	0	3	1	1	1	2	1	1	
Prophage insertion sites	t-RNA^Arg^ (*lmot17*), *comK*, between ACE3HD_13565 and ACE3HD_13990		t-RNA^Lys^ (*lmot01*), between ACN2AW_09055 and ACN2AW_09335	t-RNA^Arg^ (*lmot17*), between ACE3HG_13275 and ACE3HG_13630	t-RNA^Lys^ (*lmot01*), t-RNA^Arg^ (*lmot62*)		t-RNA^Arg^ (*lmot17*), between ACE3HN_06500 and ACE3HN_06970, t-RNA^Arg^ (*lmot62*)	comK	t-RNA^Lys^ (lmot01)	t-RNA^Lys^ (lmot01)	t-RNA^Ser^ (*lmot11*), t-RNA^Arg^ (*lmot62*),	t-RNA^Arg^ (lmot17)	comK	
ST^*a*^	ST5	ST32	ST3	ST8	ST2	ST296	ST427	ST297	ST1	ST54	ST399	ST451	ST1	
CT^*b*^	CT2789	CT6518	CT6583	CT4172	CT3336	CT6766	CT6519	CT8533	CT2752	CT4561	CT7679	CT9031	CT4246	
Isolate reference	(2)	(2)	(2)	(2)	(2)	(2)	(2)	(2)	(2)	(2)	(2)	(2)	(2)	
**Isolate ID**	**20-04347**	**21-00447**	**21-01577**	**21-04273**	**21-04322**	**21-05143**	**21-05804**	**21-05975**	**21-06248**	**21-06294**	**21-06533**	**22-03689**	**22-06838**	**23-01209**
Outbreak	Gamma9	Beta10	Psi6	sporadic	sporadic	Omikron10	Chi11	sporadic	Epsilon8	Theta11	Omega4	Epsilon13	Pi3	sporadic
Source	clinical isolate	clinical isolate	clinical isolate	clinical isolate	clinical isolate	clinical isolate	clinical isolate	clinical isolate	clinical isolate	clinical isolate	clinical isolate	clinical isolate	clinical isolate	clinical isolate
Year of isolation	2020	2021	2021	2021	2021	2021	2021	2021	2021	2021	2021	2022	2022	2023
GenBank accession no.	CP169701	CP169678	CP169680	CP169681	CP169682	CP169683	CP169685	CP169686	CP169687	CP169703	CP169688	CP169689	CP169705	CP169690
ENA accession no.	SAMEA114333441	SAMEA114333578	SAMEA114338424	SAMEA114338500	SAMEA114338501	SAMEA114338541	SAMEA114338580	SAMEA114338587	SAMEA114338608	SAMEA114338610	SAMEA114338620	SAMEA115772128	SAMEA115772129	SAMEA115772130
Sequencing method	Oxford Nanopore MinION, Illumina MiSeq	Oxford Nanopore MinION, Illumina MiSeq	Oxford Nanopore MinION, Illumina NextSeq	Oxford Nanopore MinION, Illumina NextSeq	Oxford Nanopore MinION, Illumina NextSeq	Oxford Nanopore MinION, Illumina NextSeq	Oxford Nanopore MinION, Illumina NextSeq	Oxford Nanopore MinION, Illumina NextSeq	Oxford Nanopore MinION, Illumina NextSeq	Oxford Nanopore MinION, Illumina NextSeq	Oxford Nanopore MinION, Illumina NextSeq	Oxford Nanopore MinION, Illumina NextSeq	Oxford Nanopore MinION, Illumina NextSeq	Oxford Nanopore MinION, Illumina NextSeq
Accession no. for long-read raw data	ERX11576246	ERX11576247	ERX11576248	ERX12726493	ERX11576249	ERX11576250	ERX11576252	ERX11576253	ERX11576254	ERX11576255	ERX12726494	ERX12726495	ERX12726496	ERX12726497
Number of long reads	101,745	71,889	66,174	207,696	80,636	71,613	71,114	110,129	61,748	69,163	131,719	104,733	140,767	178,894
N50 long reads	9,956	12,262	12,407	9,876	8,250	13,379	12,688	10,538	16,871	12,267	12,197	11,135	11,768	10,437
Accession no. for short-read raw data	ERX11406264	ERX11406401	ERX11412373	ERX11412449	ERX11412450	ERX11412490	ERX11412529	ERX11412536	ERX11412557	ERX11412559	ERX11412569	ERX12688901	ERX12688902	ERX12688903
Genome size (bp)	2,934,181	2,946,482	2,887,687	2,936,906	2,897,001	2,916,652	2,907,659	3,021,218	2,913,631	3,037,476	2,923,851	2,937,701	2,969,746	2,915,033
Number of short reads	1,799,810	1,555,357	3,085,456	6,177,518	5,143,410	4,080,244	4,961,316	3,909,718	6,026,918	7,759,296	4,748,972	4,786,792	2,839,004	13,239,346
GC content (%)	38	38	38	37.9	38	38	38	38	38.1	38	38	38	38	38.1
No. of protein-coding genes	2,900	2,882	2,769	2,845	2,801	2,783	2,780	2,906	2,784	3,063	2,807	2,806	2,929	2,777
No. of rRNA operons	6	6	6	6	6	6	6	6	6	6	6	6	6	6
No. of tRNA genes	67	67	67	67	67	67	67	67	67	67	67	67	67	67
Pathogenicity island(s)	LIPI-1	LIPI-1	LIPI-1	LIPI-1	LIPI-1	LIPI-1, LIPI-4	LIPI-1, LIPI-3	LIPI-1	LIPI-1, LIPI-3, LIPI-4	LIPI-1	LIPI-1	LIPI-1, LIPI-3, LIPI-4	LIPI-1, LIPI-3, LIPI-4	LIPI-1, LIPI-3, LIPI-4
Plasmid (GenBank accession no.)	CP169702 (57,553 bp)									CP169704 (86,632 bp)			CP169706	
													(55,820 bp)	
PCR serogroup	IIa	IIa	IIa	IIa	IIa	IVb	IIb	IIb	IVb	IIa	IIa	IVb-v1	IVb	IVb
Intact prophages (10)	2	2	0	1	1	0	0	2	0	2	0	0	1	0
Prophage insertion sites	t-RNA^Ser^ (*lmot11*), *comK*	*comK*, between ACE3HY_13160 and ACE3HY_13590	0	*comK*	t-RNA^Arg^ (*lmot17*)	0	0	between ACE3H5_08730 and ACE3H5_09010, between ACE3H5_13270 and ACE3H5_13700	0	t-RNA^Ser^ (*lmot11*), t-RNA^Arg^ (*lmot17*)			t-RNA^Arg^ (lmot17)	
ST^*a*^	ST7	ST18	ST26	ST412	ST29	ST388	ST224	ST59	ST4	ST101	ST222	ST382	ST217	ST219
CT^*b*^	CT9029	CT14452	CT7921	CT15626	CT15627	CT6708	CT15915	CT15925	CT8943	CT9074	CT6370	CT16568	CT5744	CT18358
Isolate reference	(2)	(2)	(2)	(2)	(2)	(2)	(2)	(2)	(2)	(2)	(2)	this work	this work	this work

^*a*^ Sequence type (ST) according to 7-locus MLST (13).

^*b*^ Complex type (CT) according to 1,701 cgMLST (14).

These genomes may promote the genetic characterization of sublineage-specific virulence, resistance, and persistence traits of *L. monocytogenes* and the tracing of outbreak clones in epidemiological studies.

## Data Availability

All genome sequences were deposited in NCBI and the European Nucleotide Archive (ENA). Accession numbers are listed in Table 1.
